# Constructing regional climate networks in the Amazonia during recent drought events

**DOI:** 10.1371/journal.pone.0186145

**Published:** 2017-10-17

**Authors:** Heng Guo, Antônio M. T. Ramos, Elbert E. N. Macau, Yong Zou, Shuguang Guan

**Affiliations:** 1 Department of Physics, East China Normal University, Shanghai, China; 2 National Institute for Space Research, São José dos Campos, São Paulo, Brazil; Beihang University, CHINA

## Abstract

Climate networks are powerful approaches to disclose tele-connections in climate systems and to predict severe climate events. Here we construct regional climate networks from precipitation data in the Amazonian region and focus on network properties under the recent drought events in 2005 and 2010. Both the networks of the entire Amazon region and the extreme networks resulted from locations severely affected by drought events suggest that network characteristics show slight difference between the two drought events. Based on network degrees of extreme drought events and that without drought conditions, we identify regions of interest that are correlated to longer expected drought period length. Moreover, we show that the spatial correlation length to the regions of interest decayed much faster in 2010 than in 2005, which is because of the dual roles played by both the Pacific and Atlantic oceans. The results suggest that hub nodes in the regional climate network of Amazonia have fewer long-range connections when more severe drought conditions appeared in 2010 than that in 2005.

## Introduction

In recent years, climate network approaches have been successfully applied to uncover some hidden interaction patterns and large scale circulation variabilities in the climate system. For instance, this approach discloses intricate tele-connections [1–3], stability properties and temporal evolutionary behavior under the influence of El Niño events [4–7]. Furthermore, some network algorithms have been proposed to forecast the early arrival of the recent El Niño [8, 9] and extreme precipitation events of monsoons over South America [10–12]. Complex networks help to trace the flow of energy and matter in the air surface temperature field [13, 14], detecting community structures [15, 16], and shedding many insights on the propagation of extreme events [17]. Network measures have shown power in differentiating regime changes from paleoclimate marine records, which covered Million years scale [18, 19]. One other example of climate network approach is to characterize different types of El Niño episodes by analyzing the evolving correlation structure of surface air temperatures [20, 21]. Furthermore, climate network analysis complements classical eigen techniques and provides additional information on the higher-order structure of statistical interrelationships in climatological data [22]. A probabilistic graphical model has been proposed to identify and to visualize network properties of the atmosphere, such as length of local memory and identification of pathways of remote impact [23].

Understanding drought events in Amazonia has fundamental importance to the stabilities of the Earth climate system because the Amazonia is one of the key tipping elements [24]. Extreme climate conditions of rainfall reductions and droughts in Amazonia have been observed in 2005 and 2010 [25–29]. The substantial difference between droughts 2005 and 2010 has been recently disclosed by computing drought period length [30]. Here we construct climate networks from the precipitation data in the Amazon region, focusing on the network structural changes under these two severe drought events in 2005 and 2010. The other motivation is that we use the Amazonian system as one example to study the stability conditions of the reconstructed networks under the influences of extreme events, which is one of the crucial questions in complex climate network analysis.

## Materials and methods

### Data

Precipitation is evaluated using the Princeton Global Forcings dataset at 0.25° resolution [31]. This dataset blends surface and satellite observations with reanalysis and is available for 1948–2010. Sea Surface Temperature (SST) is evaluated using NOAA high resolution SST data products [32]. We choose the Amazon domain as 75°*W* − 48°*W*, 5°*N* − 15°*S*, where there are 8640 spatial points. Time series for precipitation of daily resolution were built for this region, while time series for SST, are respectively, obtained by area averaging over El Niño3.0, North Atlantic (n-Atlan) and South Atlantic (s-Atlan) domains, according to the definitions of [30, 33].

Daily precipitation anomalies are calculated relative to a base period of 1961–2000. This 40-year base period is chosen as it is representative of the record of the 20th century. More specifically, given an observational series at a location (*i*, *j*) in a year (for instance, precipitation series of 2005), *y*(*t*, *i*, *j*), where *t* ∈ [1, 365], the corresponding anomaly series is computed by

$$\begin{array}{c} x\left(t,i,j\right)=\frac{y\left(t,i,j\right)-{\left\langle y\left(t,i,j\right)\right\rangle }_{T}}{\sigma_{T}}, \end{array}$$ (1)

where 〈·〉*_T_* is the average value for date *t* over *T* = 40 years from 1961–2000 and *σ*_*T*_ is the associated standard deviation. We calculate SST anomalies in the same way.

When studying the temporal variations, we compute network statistics (as explained below) based on daily anomalies with a sliding window size of 60 days and no window overlapping. In contrast, when discussing the unique characteristics of the extreme drought events in 2005 and 2010, we define summer seasons (winter) as from December to February (April to September, respectively).

### Methods

#### Expected drought period length

Recently, we have proposed to compute the drought period length *DPL*(*t*) to characterize the waiting time of the present day to the next first non-negative rainfall anomaly [30]. More specifically, from the daily rainfall anomaly series *x*(*t*) at latitude *i* and longitude *j*, we calculate *DPL*(*t*, *i*, *j*) = min{*τ*: *x*(*t* + *τ*) ≥ 0, *τ* ∈ [0, ∞)}, which captures the expecting time when anomaly series goes from negative to positive. Note that *DPL*(*t*, *i*, *j*), where *t* ∈ [1, 365] and 366 for leap years, characterizes the expectation to have a positive rain anomaly, which provides an alternative way to characterize waiting time distribution properties of the underlying processes [34].

In order to quantify the strength of dry conditions at spatial location (*i*, *j*) in a year, we count the number of dry days when *DPL*(*t*, *i*, *j*) is larger than some threshold value. Considering the specific properties of the local climate dynamics of the measurement point of the underlying grid, a fixed threshold value may not be appropriate for all locations. Therefore, we adopt a local threshold *ε*(*i*, *j*) which is chosen as 1/3 of the maximum of all the *DPL* values at the coordinate (*i*, *j*) in a year, namely, *ε*(*i*, *j*) = 1/3max*DPL*(*t*, *i*, *j*). Then, we count the number of days *N*(*i*, *j*) when *DPL*(*t*, *i*, *j*) is higher than *ε*(*i*, *j*), i.e., *N*(*i*, *j*) = #{*DPL*(*t*, *i*, *j*) ≥ *ε*(*i*, *j*)}. Qualitatively similar results have been obtained when different definitions of threshold are used, for instance, with respect to a certain quantile of the *DPL* distribution.

For better comparison of maps between different years, the *normalized drought strength* *NDS*(*i*, *j*) is introduced to normalize *N*(*i*, *j*) to the unit interval [0, 1] by

$$\begin{array}{c} NDS_{1/3}\left(i,j\right)=\frac{N\left(i,j\right)-\min_{i,j}\left(N\left(i,j\right)\right)}{\max_{i,j}\left(N\left(i,j\right)\right)-\min_{i,j}\left(N\left(i,j\right)\right)}. \end{array}$$ (2)

The subscript 1/3 of *NDS*_(_ *i*, *j*) is used to emphasize the threshold effects, which may affect the length of time intervals of the drought events. Furthermore, max_*i*, *j*_(*N*(*i*, *j*)) and min_*i*, *j*_(*N*(*i*, *j*)) are with respect to all locations (*i*, *j*). Considering the threshold effects, the *NDS*(*i*, *j*) is averaged over two more thresholds *ε*(*i*, *j*), 2/3 and 3/4 of the maximum of all the *DPL*s, namely, *NDS*(*i*, *j*) = (*NDS*_1/3_(*i*, *j*) + *NDS*_2/3_(*i*, *j*) + *NDS*_3/4_(*i*, *j*))/3.

#### Construction of climate networks

We construct the regional climate network in the Amazonian region from the precipitation anomalies with the following steps:

1. the locations (*i*, *j*) at which the considered rainfall anomaly series *x*(*t*, *i*, *j*) are used as the spatial locations of network vertices.
2. the linear Pearson correlation is computed for a relevant statistical association between any pair of series from two different locations [1, 2, 4, 6]. In addition, we restrict the analysis to the absolute values of correlation coefficients at zero lags as done previously in [13]. Note that the proper computation of correlation coefficients remains an open question [6, 35]. Nevertheless, climate networks based on statistical association measures (including linear correlation coefficient and nonlinear mutual information) provide us with novel insights for understanding the climate system, which complement the traditional eigen techniques to analyze the high dimensional spatial temporal data [22]. In consequence, a correlation matrix *CM* = *CM*_*i*, *j*_ is obtained, where *i* ∈ [1, 108], *j* ∈ [1, 80].
3. We construct the climate network by thresholding the correlation matrix *CM*_*i*, *j*_, i.e., only pairs of nodes {*i*, *j*} that satisfy *CM*_*i*, *j*_ > *γ* are regarded as linked. Using the Heaviside function *Θ*(*x*), the adjacency matrix *A*_*i*, *j*_ of the climate network is then given by
  $$\begin{array}{c} A_{i,j}=\Theta \left(CM_{i,j}-\gamma \right). \end{array}$$ (3)

Note that *A*_*i*, *j*_ inherits its symmetry from *CM*_*i*, *j*_ and the resulting climate network is an undirected and unweighted simple network.

From the viewpoint of numerical simulations, all considered measures below are based on the adjacency matrix *A*_*i*, *j*_, and hence their values necessarily depend on the choice of the threshold *γ*. So far, there is no universal threshold selection criterion to obtain *A*_*i*, *j*_. On the one hand, if *γ* is chosen too small, there are almost no linked nodes and, hence, no feasible information on the network structure of the climate system. On the other hand, if *γ* is too large, almost every node is a neighbor of every other node, which leads to numerous artifacts. Hence, we have to seek a compromise for choosing a reasonable value of *γ*. In this work, a fixed threshold *γ* is chosen such that the resulting network has a prescribed link density *ρ*, which has been widely used in climate network studies [13, 14]. A further note is that when calculating network properties using sliding windows, the threshold *γ* is chosen with respect to the first window and then it is fixed over time windows.

To characterize the resulted network *A*_*i*, *j*_, we focus on the following six network measures,

1. *link density ρ*, which measures the density of strong links that are larger than the threshold in the network, namely the density of 1 in the matrix *A*_*i*, *j*_.
2. *transitivity* T [36, 37], which characterizes the global density of closed “triangles” in the network. Note that T is sometimes referred to as the (Barrat-Weigt) global clustering coefficient.
3. *average clustering coefficient* C [38], which gives the arithmetic mean of the local clustering coefficient $C_{v}$ taken over all vertices *v*. This measure is conceptually related to, but distinct from, T. In particular, T does not explicitly take the degree of each node into account, whereas C does. In order to avoid confusion, in this work we prefer to discuss both measures separately.
4. *average path length* L, which quantifies the average geodesic (graph) distance between all pairs of vertices, i.e., the average smallest number of edges to be traversed to cover the distance between two randomly chosen vertices on the graph.
5. *assortativity coefficient* R [39], which characterizes the correlation coefficient between the degrees of all pairs of connected vertices reflecting the similarity of the connectivity.
6. *modularity* Q, which measures how good the division of the graph into modules, or how separated are the different vertex types from each other. We use the algorithms that are based on eigenvectors of the modularity matrix which has been developed by Newman [40]. The value of Q lies in the range [-1/2, 1).

Note that network measures have been widely applied to understand the climate system. In general, they have already proven to be helpful to distinguish between qualitatively different types of dynamics [18, 41–43]. The topological structures of climate networks have shown great potential for predicting the arrival of El Niño events [9], and discriminating types of El Niño and La Niña events [21]. Large scale of spatial variabilities have been obtained by networks that are reconstructed from both temperature [15, 44] and precipitation data [45], showing proper modularity structures. Here, we compute these network measures as potential candidates for discriminatory statistics, which may capture the variations of climate networks under the perturbations from extreme drought events. It remains to be a challenging task to interpret assortativity coefficient in terms of climate dynamics.

#### Improved method in constructing extreme drought networks

In order to highlight the roles of extreme drought events to the networks, we focus on the locations that are severely affected by drought as characterized by drought strength (Eq (2)). For instance, from the rectangular area of 8640 geographical points, we choose 50% locations of large *NDS*(*i*, *j*) values. In the next step, we follow the same strategy as presented in the previous section to construct extreme drought climate networks. In addition, the distributions of *NDS*(*i*, *j*) in 2005 are different with 2010, which yields different sets of locations in these two years (as will be shown later). Therefore, we show the network measures separately.

In addition to the extreme drought networks reconstructed from the locations that are characterized by top *NDS* values, similar networks obtained from small *NDS* values (say, 50% quantile of the distribution of *NDS* values) provide complement insights for the understanding of climate without drought conditions. When discussing about the choice of regions of interest, we compare the results from extreme drought networks and that are obtained from networks reconstructed from small *NDS* values.

#### Delay effects by surrounding oceans to the Amazonia

The surrounding Pacific and Atlantic oceans play crucial roles in the formation of precipitation networks in the Amazonia. Therefore, we interpret the unique network properties during the droughts by studying the ocean effects. More specifically, we extract time delay effects from the ocean to the precipitation patterns in Amazonia by means of transfer entropy, which has been recently proposed in [46]. This approach was based on the ordinal patterns of time series data and the advantage is that it allows to extracting time delays with improved statistical power. Using this approach, we identify the delay effects between ENSO (El Niño 3.0 series), north Atlantic and south Atlantic to the region of interest in the Amazonia. Given the precipitation anomaly series *x*(*t*) spatially averaged over the Amazon region and *S*(*t*) of SST, we compute the delayed mutual information MI(τ) by shifting MI(t-τ) in order to extract the delay information of the oceans. The delays have shown 95% of significance level by surrogate testing [46].

## Results

### General behavior of drought period length

**Fig 1:**
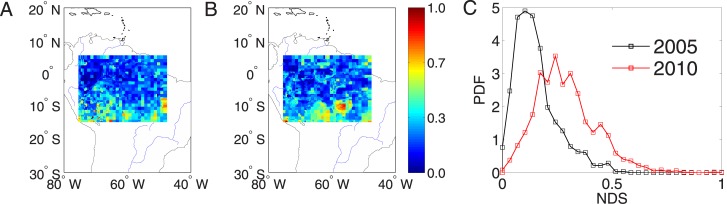
Normalized drought strength over Amazon region. (A) 2005, (B) 2010, and (C) probability distribution functions of normalized drought length.

The spatial distributions of normalized drought strength of the year 2005 and 2010 in the Amazonian region are respectively shown in Fig 1A and 1B. In 2005, the center of much severe dry conditions is observed in the southwestern region, however, the severe dry conditions in 2010 are shifted to the center of the Amazonian area. In order to show the difference between these two years, we summarize the results by probability density functions (PDF) of *NDS* as shown in Fig 1C. The PDF is skewed to the left in 2005 indicating less severity comparing to 2010. In conclusion, in 2010, the Amazonia experienced a large spatial coverage of long temporal severe dry conditions.

### Seasonal variabilities of network measures

**Fig 2:**
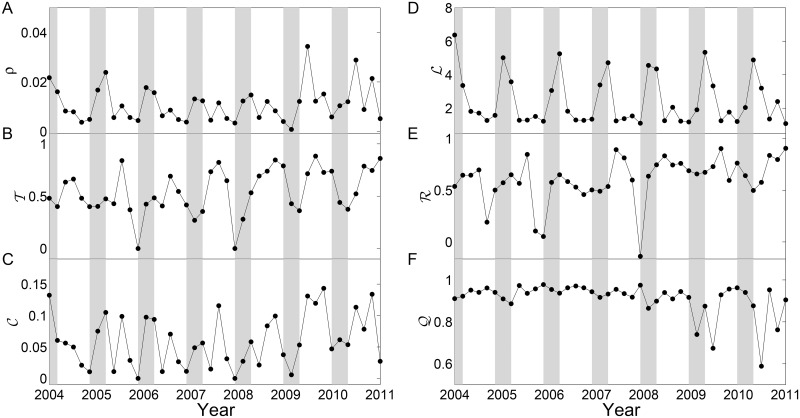
Network measures and gray areas highlight the austral summer periods. (A) link density *ρ*, (B) transitivity T, (C) clustering coefficient C, (D) average path length L, (E) assortativity R and (F) modularity Q. Gray time windows highlight the summer seasons.

We construct climate networks with running windows of 60 days (no window overlapping). We find that window sizes ranging from 30 to 90 days do not alter our results substantially. In addition, the threshold *γ* is chosen according to a specific link density of *ρ* = 0.01 for the first window and then this value is fixed when sliding windows over time. We follow the traditional choices of these parameters when constructing climate networks using sliding window techniques [20]. Then, we compute the network measures ρ,T,C,L,R and Q for each window, such that following the variation over time. There are 8640 nodes in each network. It is a well-known fact that the network measures depend on the choice of threshold value *γ*. To consider these possible effects, the results shown in Fig 2 are averaged over three representative threshold values such that the link densities of the first window are *ρ* = 0.01, 0.02, and 0.03.

We find that network measures ρ,T,C,L oscillate regularly, showing seasonal effects. For instance, the average path length L reaches a maximum in the summer rainy seasons while the minimum in the dry winter seasons (Fig 2D). This is because the network as we built becomes sparser in the summers requiring large path length to reach different parts of the network, which is consistent with *ρ* (Fig 2A), T (Fig 2B) and C (Fig 2C). In contrast, we noticed that T reached minima during summer periods (Fig 2B). However, the seasonal effects are not well captured by R and Q (Fig 2E and 2F). In particular, Q shows big variations after 2009, while almost invariant before 2009. This indicates that the possible subgraph structures are not pronounced in 2009 and 2010 than that in other years. The underlying mechanisms of the variations of network measures remain to be investigated in terms of the regional climate dynamics.

### Seasonal variabilities of extreme drought networks

**Fig 3:**
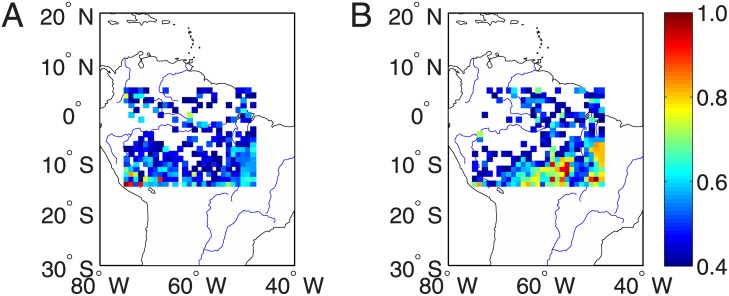
Distribution of 50% large values of drought strength across the Amazon region in the year (A) 2005, and (B) 2010.

In order to clarify the construction of extreme drought networks, we show in Fig 3 the locations that are experienced large drought strength, i.e., 50% of top values across the entire region. In other words, 50% of relative small values of *NDS* are suppressed in the color plot. It is easy to see that our method highlights the roles of locations of extreme drought events. Different definitions of top *DPL* values yield different sizes for the reconstructed extreme drought networks. Therefore, we repeat the same analysis for different percentages (thresholds from 30% to 70%) of top *NDS* values. When more locations of top *NDS* values are included in the extreme networks, the results are close to the case of Fig 2.

**Fig 4:**
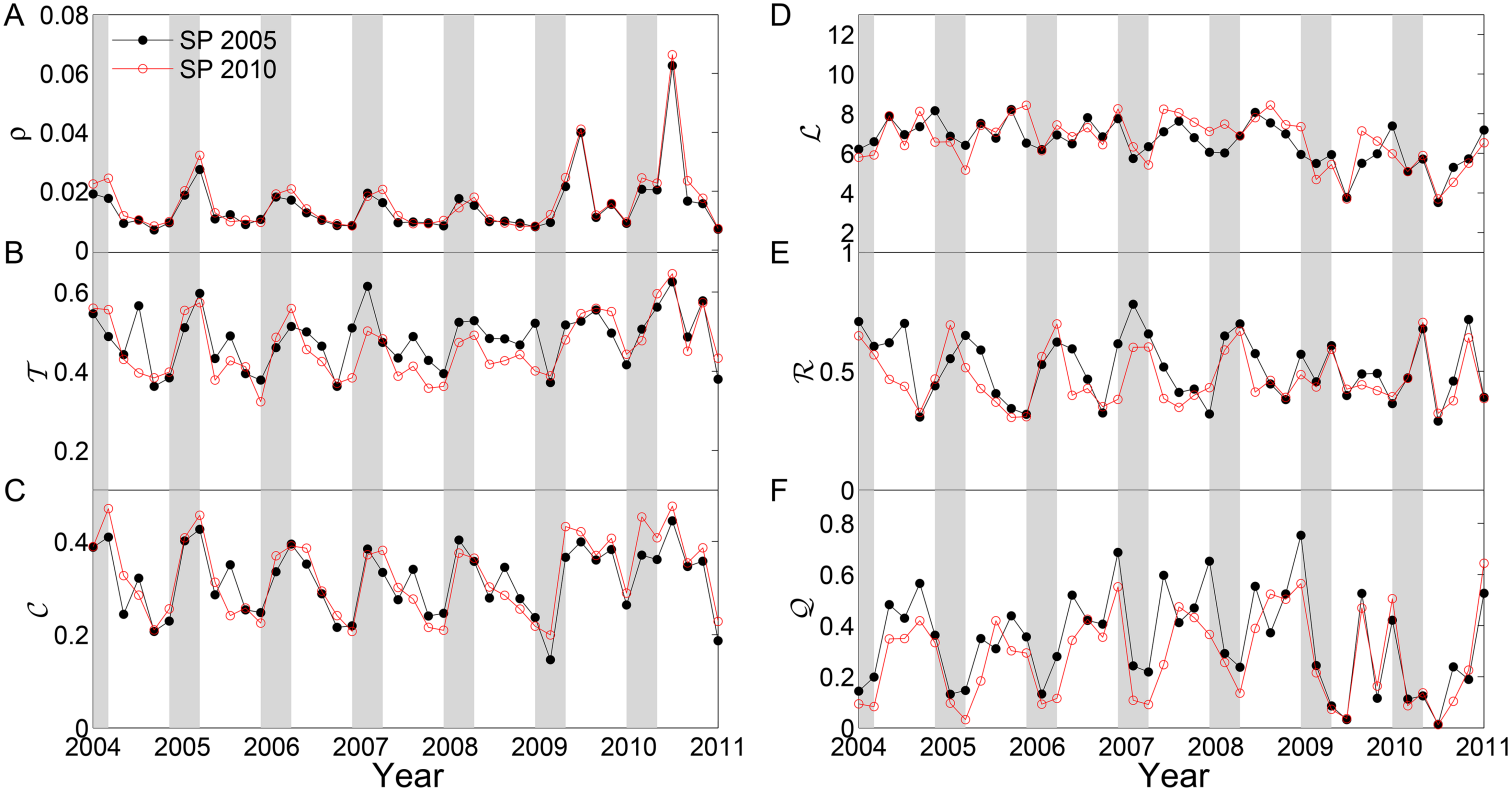
Network measures of extreme drought networks. Gray areas highlight the austral summer periods (• spatial points of 2005, and ° spatial points of 2010). (A) link density *ρ*, (B) transitivity T, (C) clustering coefficient C, (D) average path length L, (E) assortativity R and (F) modularity Q. Gray time windows highlight the summer seasons.

In the next step, we follow the same strategy as in the previous section to show the variations of network measures over time (Fig 2). Note that there are different sets of locations in the resulting networks, which lead to two curves in Fig 4. One curve corresponds to the spatial points of the year 2005 and the other is for 2010. Comparing Fig 4 with Fig 2, rather similar temporal variations of network measures have been obtained for the extreme drought networks. Some amount of difference at different time windows may be discerned, which however requires careful interpretations and statistical evaluations in the future work.

### Regions of interest defined by large network degrees

**Fig 5:**
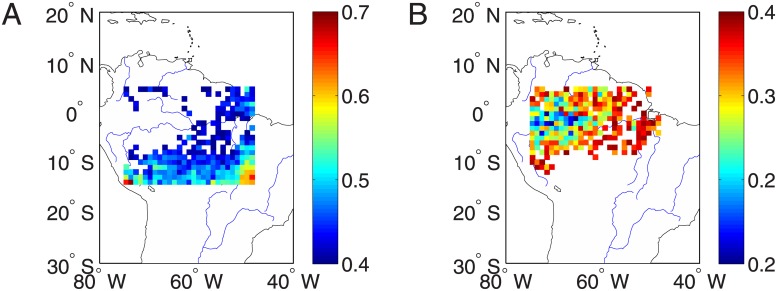
Maps of averaged *NDS* values. Locations of (A) 50% upper quantile and (B) 50% lower quantile of *NDS* values.

In order to disclose the particular network features for the drought events in 2005 and 2010, we focus on the correlation structures to some particular regions of interest under the extreme drought conditions. Since the sets of locations of top *NDS* values are different between 2005 and 2010 as shown in Fig 3, we cannot compare the networks directly. Therefore, we first compute the map of *NDS* values for each year from 2004 to 2010 and then produce the map of averaged *NDS* values, which characterizes the general behavior of drought period length in the Amazonia. From this map, we choose top 50% of locations of large *NDS* values to construct extreme drought networks and the chosen locations are shown Fig 5A. In contrast, drought networks obtained from 50% of locations of small *NDS* values capture the climate conditions without serious droughts and the locations are shown Fig 5B.

**Fig 6:**
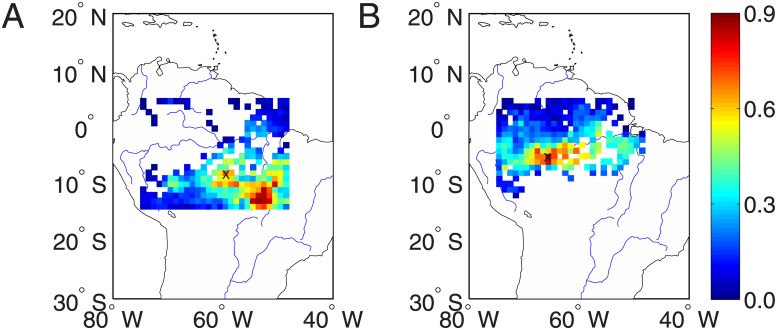
Map of the averaged network degrees (normalized to unit interval) for locations that are characterized by 50% upper quantile of *NDS* values (A), and (B) 50% lower quantile of *NDS* values. The ROIs are denoted by ×.

In the next step, we construct networks for these two different sets of locations. More specifically, given precipitation anomaly time series of the chosen locations (for instance, locations of 50% upper quantile of *NDS* values), we construct the corresponding climate networks based on the map of the averaged correlation matrix *CM*_*i*, *j*_ over seven years from 2004 to 2010. The map of network degrees is shown in Fig 6A and the threshold value *γ* is chosen such that the network link densities *ρ* = 0.01. We have obtained similar results when varying the threshold.

In a full analogy, the map of network degrees of locations of 50% lower quantile of *NDS* values is shown in Fig 6B. On average, the area of large network degrees of extreme drought network from 50% upper quantile of *NDS* values spread in the Southeast Amazon (Fig 6A). In contrast, the area of large network degrees of normal drought conditions from 50% lower quantile of *NDS* values is more concentrated in the central region (Fig 6B).

Comparing Fig 6 to Fig 1, we find some relationship between the map of averaged network degrees and *NDS*, which may provide insights for choosing proper regions of interest. Neglecting the boundary points in Fig 6A, in general, the central and south east regions where experienced larger drought strength have large network degrees in networks of both extreme droughts and normal climate conditions without drought events. Therefore, based on Fig 6, we identify the network hub as Regions of Interest (ROIs) for further correlation analysis in the next section. In particular, the location at (58°*W*, 8.5°*S*) is identified as the ROI of the extreme drought networks, which is pinpointed as a cross mark in Fig 6A. Similarly, the hub region at (65°*W*, 5.5°*S*) in Fig 6B is identified as the ROI for the normal climate conditions without extreme drought events.

### Correlations to the ROIs

**Fig 7:**
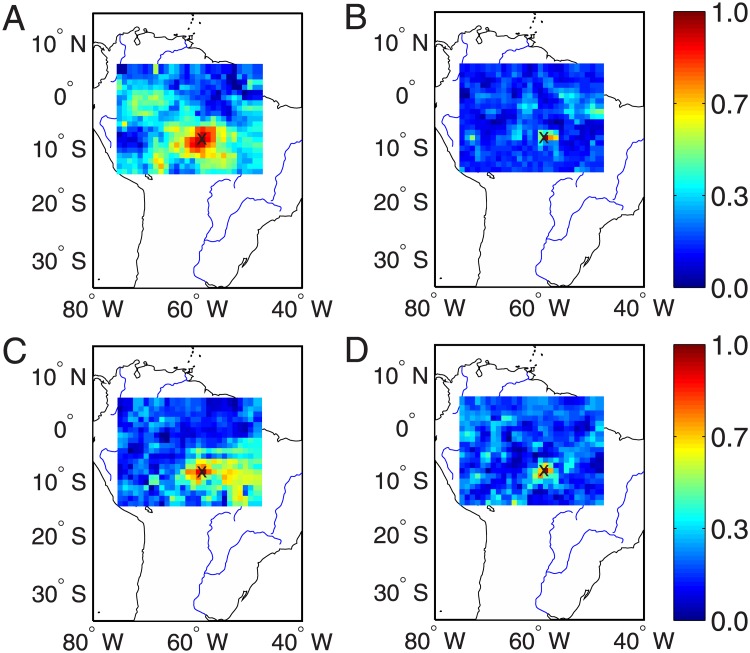
Map of correlation coefficients to the ROI at (58°*W*, 8.5°*S*) as indicated by ×, where large network degrees are observed in the extreme drought networks. (A) austral summer 2005, (B) winter 2005, (C) summer 2010, and (D) winter 2010.

**Fig 8:**
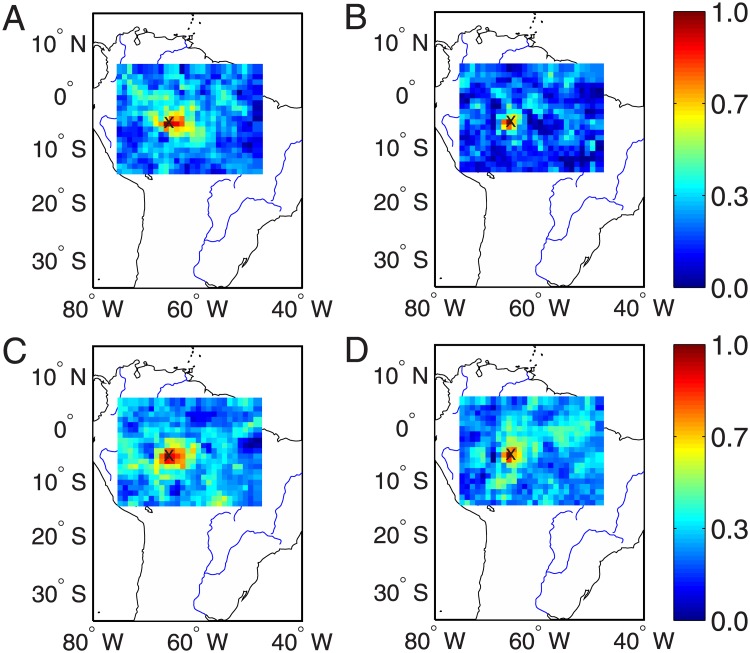
Map of correlation coefficients to the ROI at (65°*W*, 5.5°*S*) as indicated by ×, where large network degrees are observed in the network of normal drought conditions. (A) austral summer 2005, (B) winter 2005, (C) summer 2010, and (D) winter 2010.

Computing network measures (as shown in Figs 2 and 4) mainly show seasonal variations. The difference between the recent two severe droughts in 2005 and 2010 requires further analysis. In the next step, we focus on the properties of connectivity to the ROIs and the results are shown in Figs 7 and 8. In addition, we compute the connectivity for the rainy and dry seasons separately to show the difference in the connectivity patterns.

In the discussion below, we take the ROI at (58°*W*, 8.5°*S*) as an example. The consistent results have been obtained for the ROI at (65°*W*, 5.5°*S*) which is chosen for the climate conditions without extreme drought events. The computation of correlations to the ROI of Fig 7 is the following: We first draw squares using the ROI as the concenter on the map and the distance between two squares has the spatial resolution of 0.25° which is determined by the resolution of the grid data. Next, the correlations to the ROI of all locations along each square are respectively computed and then an averaged value over square is used for the *y*-axes, while the distance of the square to the concenter of ROI is for the *x*-axes of Fig 7. In addition, when computing the correlation coefficients between the ROI and the locations along the concentric square, we calculate the standard deviation values to represent the significance level. We note that more advanced methods are required to make statistical tests, for instance, by using surrogate techniques. However, it remains open to create appropriate surrogate data which are helpful for spatial temporal data (i.e., precipitation). This line of research needs careful algorithmic steps, which is beyond the scope of the current work and will be of future work.

**Fig 9:**
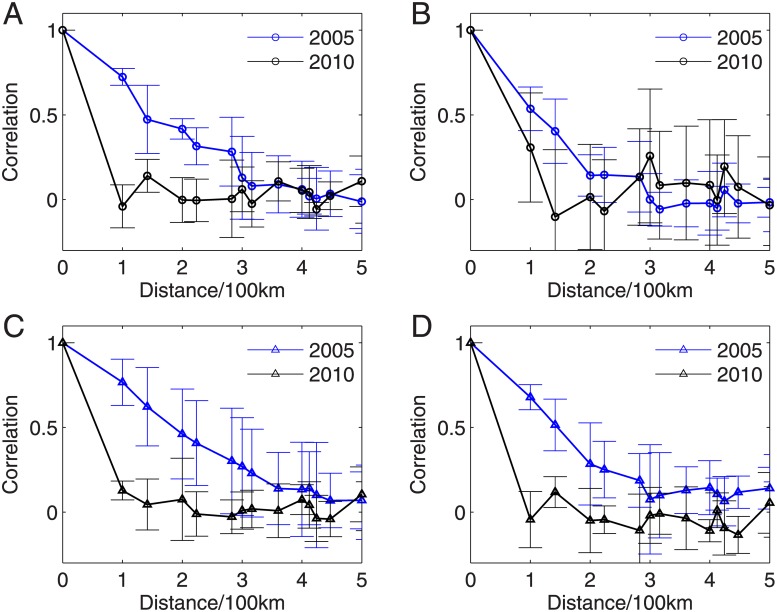
Correlation distances to the ROI at (58°*W*, 8.5°*S*) in the extreme drought network (A, B), and respectively, to the ROI at (65°*W*, 5.5°*S*) in the network of normal drought conditions (C, D). (A, C) summer, and (B, D) winter. Error-bars correspond to the standard deviations.

Because of the strong correlation between two geographical neighboring locations, the correlations to the ROI show gradual decays when moving away from the ROI [47–49]. We find some difference between 2005 and 2010, especially the summer seasons in both years. Comparing Fig 7A to Fig 7C, we find that the correlations to the ROI show relative slower decays in 2005 than that of 2010. This decaying rate has been summarized in Fig 9A in terms of the geographical distances. Correlations to the ROI drop to less than 1/*e* (≈ 0.36) when the distance to the ROI is larger than 300km in the year 2005, in contrast, this drop appears when the distance is less than 50km in 2010. However, in both winter seasons, the correlations to ROI show much faster decreasing speeds as shown in Fig 7B and 7D. Nevertheless, the spatial correlations show faster-decays in the winter season 2010 than that in 2005 (shown in Fig 9B).

Qualitatively similar conclusions are obtained when performing the correlation analysis to the ROI at (65°*W*, 5.5°*S*), which are shown in Figs 8, 9C and 9D. In summary, the relative faster decays of the cross-correlation coefficients suggest that hub nodes of the reconstructed precipitation networks in the Amazonia have fewer long-range connections in 2010 than that in 2005.

### Correlations to surrounding oceanic SST

We conjecture that the observation that there are fewer long-range connections in 2010 might come from the distinct roles of the surrounding oceans. In particular, both the Pacific and Atlantic had played different roles in developing the precipitation patterns in Amazonia in 2005, and respectively, 2010.

The anomalous rainfall reduction in the Amazonia has been related to the anomalous warming of the SST in the surrounding oceans [30], however, with different delays from the Pacific to the Atlantic [46]. The roles played by the oceans are associated with the climate conditions of the precipitation network, thus the connectivity properties.

**Fig 10:**
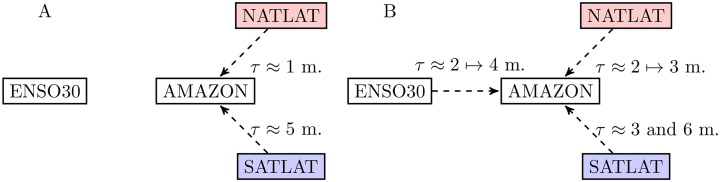
Delays of the ocean effects on the precipitations in the Amazon. (A) 2005, no significant delay effects have been identified from the ENSO region, and (B) 2010.

To this end, we show the delays of the ocean’s effects in Fig 10. Note that the algorithmic details of extracting time delays and the associated statistical significance are presented in [46]. In 2005, no significant effects by the ENSO region have been observed (Fig 10A). We identify one month delay of the north Atlantic and, respectively, 5 months delay of the south Atlantic to the precipitation patterns in the Amazonia. In contrast, significant influences by both the ENSO (from 2 to 4 months delays) and Atlantic Oceans (from 2 to 3 months by the north and 3 and 6 months by the south) have been identified in 2010 (Fig 10B). Note that the delays have shown 95% of significance level by surrogate testing [46].

## Discussions and conclusions

In summary, we have reconstructed the regional climate network in the Amazonia from the precipitation data, focusing on the associated stability conditions under the recent two severe drought events in 2005 and 2010. In addition, we have constructed extreme drought network from spatial locations that are severely affected by drought events, comparing to the networks of normal climate conditions without droughts.

Generally speaking, the network measures (i.e., link density, clustering coefficient, transitivity, average path length, assortativity and modularity etc.) show some annual variations (distinctions between seasons). In order to disclose the particular changes in the Amazonian region, we focus on the connectivity to the regions of interest (ROIs). The ROI at 58°*W*, 8.5°*S* has been chosen because it has large network degrees in the extreme drought networks, which means it has experienced much longer drought period length during the extreme climate conditions in 2005 and 2010. In addition, the ROI at 65°*W*, 5.5°*S* has large network degree in the network of normal drought conditions. Two geographically close regions show gradual (trivial) decays in the correlation structures to the particular chosen ROIs. In the presence of extreme drought events, we have found that the connectivity to the ROIs shows much faster-decaying speed during the drought event 2010 than that of 2005. This suggests that hub nodes in the reconstructed precipitation network of 2010 in the Amazonia have fewer long-range connections that of 2005. We have conjectured the difference between the 2005 and 2010 connectivity patterns to the ROIs has been related to the role played by both the Pacific and Atlantic Oceans.

One interesting direction for future work is to construct interacting networks, for instance, a network of networks from the Amazon, Pacific and Atlantic Oceans. The approach of network of sub-networks may provide many detailed changes of the coupling patterns among these three different geological regions [50], following the propagation of extreme events [17].
